# *In Vivo* Availability of Pro-Resolving Lipid Mediators in Oxazolone Induced Dermal Inflammation in the Mouse

**DOI:** 10.1371/journal.pone.0143141

**Published:** 2015-11-23

**Authors:** Julia Homann, Jing Suo, Mike Schmidt, Natasja de Bruin, Klaus Scholich, Gerd Geisslinger, Nerea Ferreirós

**Affiliations:** 1 *pharmazentrum frankfurt*/ZAFES, Institute of Clinical Pharmacology, Goethe-University, Frankfurt, Germany; 2 Fraunhofer Institute for Molecular Biology and Applied Ecology IME, Project Group TMP, Frankfurt, Germany; Universidade Federal do Rio de Janeiro, BRAZIL

## Abstract

The activation and infiltration of polymorphonuclear neutrophils (PMN) are critical key steps in inflammation. PMN-mediated inflammation is limited by anti-inflammatory and pro-resolving mechanisms, including specialized pro-resolving lipid mediators (SPM). We examined the effects of 15-epi-LXA_4_ on inflammation and the biosynthesis of pro-inflammatory mediators, such as prostaglandins, leukotriene B_4_ and various hydroxyeicosatetraenoic acids and SPM, in an oxazolone (OXA)-induced hypersensitivity model for dermal inflammation. 15-epi-LXA_4_ (100 μM, 5 μL subcutaneously injected) significantly (P < 0.05) reduced inflammation in skin, 24 hours after the OXA challenge, as compared to skin treated with vehicle. No significant influence on the biosynthesis of prostaglandins or leukotriene B_4_ was observed, whereas the level of 15S-hydroxy-eicosatetraenoic acid was significantly (P < 0.05) lower in the skin areas treated with 15-epi-LXA_4_. In spite of the use of a fully validated analytical procedure, no SPM were detected in the biological samples. To investigate the reason for the lack of analytical signal, we tried to mimic the production of SPM (lipoxins, resolvins, maresin and protectin) by injecting them subcutaneously into the skin of mice and studying the *in vivo* availability and distribution of the compounds. All analytes showed very little lateral distribution in skin tissue and their levels were markedly decreased (> 95%) 2 hours after injection. However, docosahexaenoic acid derivatives were biologically more stable than SPM derived from arachidonic acid or eicosapentaenoic acid.

## Introduction

Specialized pro-resolving endogenous lipid-mediators (SPM) form a new class of molecules, for which key roles in the counter-regulation of inflammation and consequently resolution have been established in recent years [1, 2]. SPM are derived from different polyunsaturated fatty acids (PUFAs) and include metabolites of arachidonic acid (AA) (e.g. LXA_4_ and LXB_4_), docosahexaenoic acid (DHA) (e.g. D-series resolvins, maresins and protectins) and eicosapentaenoic acid (EPA) derivatives (e.g. E-series resolvins and LXA_5_). Inflammation is involved in the pathophysiological processes of many diseases, such as rheumatoid arthritis [3, 4], periodontitis [5], asthma bronchiale [1, 6], cardiovascular diseases [2, 7], Alzheimer’s disease [8] and diabetes [7, 9]. The discovery of the SPM might lead to novel targets and drugs for the pharmacological treatment of inflammatory diseases, based on a detailed characterization of the biochemical pathways leading to resolution. The biosynthesis and positive effects of the SPM have already been established for different animal disease models, such as peritonitis [10–14], colitis [15–18], asthma [19, 20] and atherosclerosis [21].

With regard to skin diseases, most investigators have studied the role of lipoxin analogues in acute skin inflammation models [22–24], with the exception of Schottelius et al., who additionally examined the influence of a stable LXA_4_ analogue in a trimellitic anhydride-induced delayed type hypersensitivity model [25], showing the effectiveness of this compound in reducing edema formation and cell infiltration. However, its influence on the synthesis of SPM and other lipid-mediators related to the inflammatory process were not examined.

Here, we determined SPMs in dermal inflammation using LC-MS/MS and studied the effect of 15-epi-LXA_4_ in a delayed type hypersensitivity (DHT) model. In this model, an in vivo T cell–dependent immune response is manifested as an inflammatory reaction that reaches peak intensity 24 to 48 h after antigenic challenge. The DTH reaction occurs at the site of antigen deposition; the skin serves as the usual site for eliciting DTH in experimental systems. Measuring the intensity of DTH involves quantitating some aspect of the local inflammatory response, which can be used to follow the activity of immunosuppressive molecules and/or suppressor T cells *in vivo*. Typical sites of challenge are the footpad or the ears [26, 27]. In addition, the availability, distribution and stability of SPMs was studied *in vivo* after subcutaneous injection of the SPMs.

The biological effects of 15-epi-LXA_4_ on the oxazolone (OXA)-induced skin inflammation model and on non-invasive, highly sensitive bioluminescence imaging (BLI) of myeloperoxidase (MPO) activitiy [22, 25] were also determined. 15-epi-LXA_4_, which was reported to be biosynthesized by acetylated or nitrosylated cyclooxygenase-2 (COX-2) or cytochrome P450 (CYP450)-enzymes, was administered subcutaneously to mimic its biosynthesis in dermal inflammation. In contrast, other studies have pursued a therapeutic approach by the topical application of chemically modified, stable lipoxin analogues. Zhang et al. used LXA_4_ in their studies on the influence of this mediator on acute skin inflammation, but it was injected intraperitoneally [24]. To study the influence of SPM (in this case 15-epi-LXA_4_) in the context of dermal inflammation, not only was this class of determined compound, but also other inflammatory mediators, such as prostaglandins, LTB_4_ and several HETEs. To our knowledge, this is the first report in which the interactions of 15-epi-LXA_4_ and other inflammatory mediators have been studied in the physiological context of dermal inflammation.

For *in vivo* determination of SPM in skin, a chiral LC-MS/MS analytical method has been fully validated. To investigate the reason for the lack of positive results during the determination of SPM, a comparative situation was induced by injecting the analytes subcutaneously into the back of the mice. For these analytes, tissue distribution and *in vivo* availability were assessed after injection.

## Materials and Methods

### Animals

C57BL/6N mice, 9 weeks old, male, were obtained from Charles River (Sulzfeld, Germany) and maintained under controlled conditions: Upon arrival, the animals were housed in Type II cages on sawdust bedding. The room was illuminated by a 12 h light-dark cycle (on 7am, off 7pm), the temperature range was 20°C to 22°C and the relative humidity range was 30% to 40%. The mice were kept in a rodent holding room prior to transfer to the test room for the study. They were allowed to acclimatize for at least 7 days before the start of the experiment and had free access to food and tap water. All procedures described were approved by the local Ethics Committee for Animal Research of the Regierungspräsidium Darmstadt (Darmstadt, Germany) and adhered to the guidelines of GV-SOLAS for animal welfare in science.

### Influence of 15-epi-LXA_4_ in skin inflammation

#### Oxazolone-induced dermatitis model

**Fig 1:**
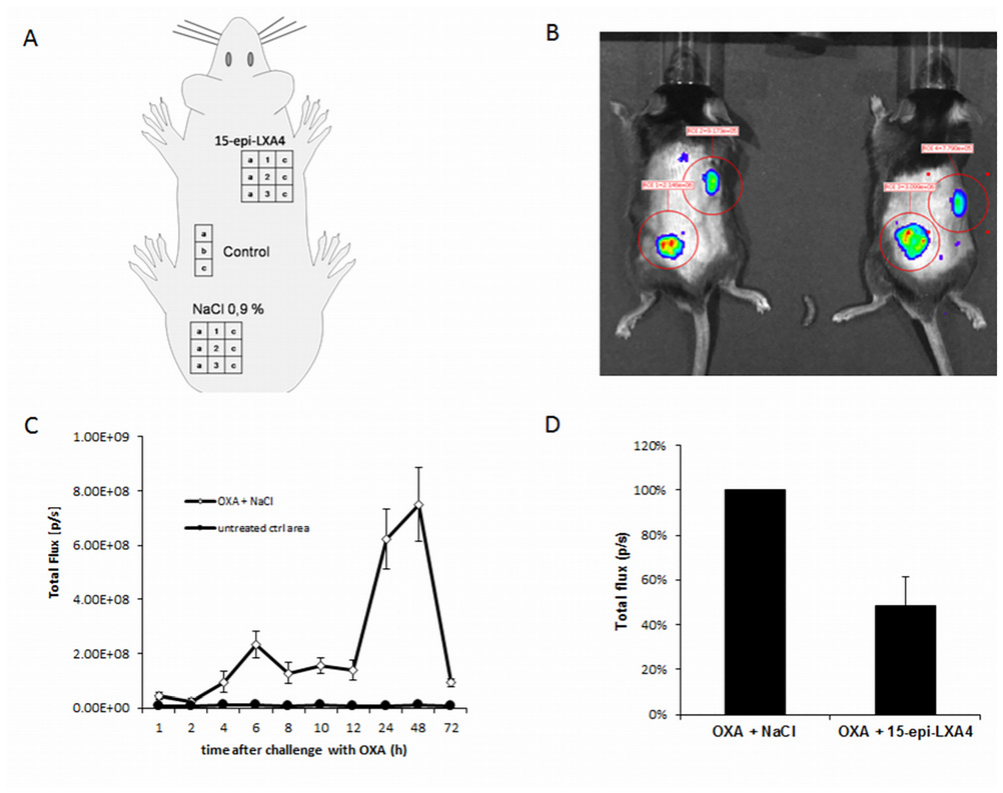
Influence of 15-epi-LXA_4_ on bioluminescence signals in dermal inflammation. Schematic division of the shaved back of mice as the experimental setup (A) and with corresponding bioluminescence images (B). Results of the bioluminescence imaging show a time course of signal intensity (C) and the signal intensity at 24 h after OXA-challenge (D).

Mice (n = 5) were sensitized by a single epicutaneous application of 100 μL of 3% 4-Ethoxymethylene-2-phenyl-2-oxazolin-5-one (oxazolone, OXA), dissolved in acetone/olive oil (3:1 v/v), all purchased from Sigma-Aldrich Chemie GmbH (Munich, Germany), to the shaved abdomen (sensitization phase). Seven days later, 5 μL of 1% OXA, again dissolved in acetone/olive oil (3:1 v/v) was applied to the middle of two measured areas (12 x 12 mm) on the shaved back (challenge phase). The areas were divided into three sections (1–3) (4 x 12 mm) each one of which was further subdivided into three subsections (a-c), resulting in 9 equal squares, 1a-1c, 2a-2c, 3a-3c (4 x 4 mm each). For the determination of the effect of 15-epi-LXA_4_ in the OXA-induced dermatitis model, ten minutes after the application of OXA, 5 μL of 100 μM 15-epi-LXA_4_ in saline solution (0.9% NaCl) was injected subcutaneously into the middle of the test area (square 2b). Five μL 0.9% NaCl were injected subcutaneously into the other (square 2b). Additionally, a third area (4 x 12 mm) was defined and subdivided into 3 equal squares (4 x 4 mm each). This control area remained completely untreated (Fig 1A)

#### Bioluminescence imaging

For the measurement of MPO, an enzyme present in PMN and therefore used as a marker for PMN infiltration, BLI was employed, as described previously [28]. Here, we used IVIS 200 bioluminescence imaging system (f/stop, 1; no optical filter) and Living image software version 4.3.1 (Caliper Life Sciences, Mainz, Germany). Twenty-four hours after the injection of 15-epi-LXA_4_ or 0.9% NaCl, mice received 5-amino-2,3-dihydro-1,4-phthalazinedione (Luminol sodium salt), purchased from Sigma-Aldrich Chemie GmbH (Munich, Germany), dissolved in phosphate buffered saline (PBS), obtained from PAA Laboratories (Pasching, Austria), to a final concentration of 50 mg/mL, (200 mg/kg BW, i.p.). Under anesthetic (isoflurane inhalation) and after 20 minutes, mice were imaged (exposure time, 60 sec; binning, 8; field of view (FOV), 13.2 cm). Regions of interest (ROI) were manually selected over the application sites. The total photon emissions (radiance) were determined. Total flux (photons/sec) is defined as the radiance (photons/sec/cm^2^/steradian) in each pixel summed or integrated over the ROI area (cm^2^) x 4π. A time course study of OXA-induced infiltration of PMN was conducted by measuring the radiance at 1, 2, 4, 6, 8, 10, 12, 24, 48 and 72 h after challenge in the OXA-treated and the untreated control area. The influence of 15-epi-LXA_4_ on inflammation was determined after 24 h, since the MPO activity was maximal at that time point. The ROI consisted of the two specific areas (12 x 12 mm) into which NaCl or 15-epi-LXA_4_ were injected. After imaging, mice were killed and the 9 measured skin squares of each treated area, in addition to the 3 control skin squares of the untreated area, were taken separately and snap frozen directly in liquid nitrogen and stored at -80°C until further analysis with LC-MS/MS.

#### Determination of COX-2 expression using qPCR

10 minutes after challenge, a 3 mm^2^ piece of skin was removed from 5 sensitized and 5 non-sensitized mice. RNA was extracted from the skin of each animal using the Peqlab Precellys RNA Tissue Kit (Erlangen, Germany). The RNA was then transcribed into cDNA using the Thermo Scientific First Strand cDNA Synthesis Kit (Dreieich, Germany). The expression of Cox-2 as well as two housekeeping genes, PPIA and RPL13a, were measured in a quantitative real-time PCR using the Bio&Sell 5x QPCR EvaGreen Mix (Feucht, Germany) and the BioRad CFX96 Touch real-time PCR detection system (Munich, Germany). Statistical analysis was done in IBM’s SPSS using the Mann-Whitney-U test. Results were expressed as means ± SEM.

#### Quantitation of prostanoids and HETE with LC-MS/MS

Determination of pro-inflammatory mediators was conducted as previously published [29]. Briefly, for sample preparation, a LLE-procedure was used. Homogenated tissue was extracted twice with 600 μL of ethyl acetate (VWR BDH Prolabo, Wien, Austria) and the combined organic layers were removed under a nitrogen stream at 45°C. For the chromatographic separation of 5S-hydroxy-6E,8Z,11Z,14Z-eicosatetraenoic acid (5-HETE), 15S-hydroxy-5Z,8Z,11Z,13E-eicosatetraenoic acid (15-HETE) and 5S,12R-dihydroxy-6Z,8E,10E,14Z-eicosatetraenoic acid (leukotriene B4, LTB_4_), a Gemini NX C18 column and precolumn were used (150 x 2 mm ID, 5 μm particle size, and 110 Å pore size; Phenomenex, Aschaffenburg, Germany). A linear gradient was used at a flow rate of 0.5 ml/min with a total run time of 17.5 min. Mobile phase A consisted of water:ammonia (100:0.05, v/v), and mobile phase B, of acetonitrile:ammonia (100:0.05, v/v). 20 μL were injected into the LC-MS/MS system.

The chromatographic separation of prostanoids, 9-oxo-11R,15S-dihydroxy-5Z,13E-prostadienoic acid (prostaglandin E2, PGE_2_), 9S,15S-dihydroxy-11-oxo-5Z,13E-prostadienoic acid (PGD_2_), 9S,11R,15S-trihydroxy-5Z,13E-prostadienoic acid (PGF_2α_), 6-oxo-9α,11α,15S-trihydroxy-prost-13E-en-1-oic acid (6-keto-PGF1α) and 9S,11,15S-trihydroxy-thromboxa-5Z,13E-dien-1-oic acid (thromboxane B_2_, TXB_2_) was achieved using a Synergi Hydro-RP column (150 x 2 mm ID, 4 μm, Phenomenex, Aschaffenburg, Germany) and a precolumn of the same material. Chromatographic separation was carried out in gradient elution mode at a flow rate of 0.3 ml/min. Total run time was 16 min. Mobile phase A consisted of water/formic acid (100:0.0025, v/v) and mobile phase B, of acetonitrile/formic acid (100:0.0025, v/v).

### Availability of SPM in mice after subcutaneous injection

#### Determination of the stability and the distribution of SPM in mice skin

Mice (n = 9) were anesthetized by the injection (i.p.) of 150 μL of a solution consisting of 4.5 mL 0.9% NaCl, 0.25 mL xylazine (10 mg/kg BW) and 1 mL ketamine (100 mg/kg BW) and their backs were shaved two days before starting the experiment. The back was divided into 9 sections 1–9 (4 x 12 mm) of which each was further subdivided into three subsections (a-c), resulting in 27 equal squares 1a, 1b, 1c–9a, 9b, 9c (4 x 4 mm each). Five μL saline solution containing LXA_4_, 15-epi-LXA_4_, 6-epi-LXA_4_, LXB_4_, LXA_5_, 17-epi-RvD1, RvD1, RvD2, as well as 7-MaR1 and PDx each at a concentration of 10 μM, were injected subcutaneously into the middle of the measured area (square 5b) (see section “Stability and Distribution of SPM in murine skin after subcutaneous injection” for more information). For the determination of the availability and distribution of the SPM, mice were killed (carbon dioxide inhalation) directly after injection (n = 3), 30 minutes after injection (n = 3) and 2 hours after injection (n = 3). The 27 measured skin squares were collected separately and snap frozen directly in liquid nitrogen and stored at -80°C until analysis with LC-MS/MS.

### Quantitation of SPM with LC-MS/MS

5S,6R,15S-trihydroxy-7E,9E,11Z,13E-eicosatetraenoic acid (lipoxin A_4_, LXA_4_), 5S,6R,15R-trihydroxy-7E,9E,11Z,13E-eicosatetraenoic acid (Aspirin^®^-triggered lipoxin A_4_, 15-epi-LXA_4_), 5S,6S,15S-trihydroxy-7E,9E,11Z,13E-eicosatetraenoic acid (5(S), 6(S), 15(S)-lipoxin A_4_, 6-epi-LXA_4_), 5S,14R,15S-trihydroxy-6E,8Z,10E,12E-eicosatetraenoic acid (LXB_4_), 5S,6R,15S-trihydroxy-7E,9E,11Z,13E,17Z-eicosapentaenoic acid (LXA_5_), 7S,8R,17R-trihydroxy-4Z,9E,11E,13Z,15E,19Z-docosahexaenoic acid (Aspirin^®^-triggered resolvin D1, 17-epi-RvD1), 7S,8R,17S-trihydroxy-4Z,9E,11E,13Z,15E,19Z-docosahexaenoic acid (RvD1), 7S,16R,17S-trihydroxy-4Z,8E,10Z,12E,14E,19Z-docosahexaenoic acid (RvD2), as well as 7*R*,14*S*-dihydroxy-4Z,8*E*,10*E*,12*Z*,*16Z*,*19Z*-docosahexaenoic acid (7-maresin 1, 7-MaR1) and 10S,17S-dihydroxy-4Z,7Z,11E,13Z,15E,19Z-docosahexaenoic acid (PDx), all purchased from Cayman Chemicals (Tallin, Estonia), were quantified as previously described [30]. The analytical method published was modified to include 7S,16R,17S-trihydroxy-4Z,8E,10Z,12E,14E,19Z-docosahexaenoic acid-21,21’,22,22,22-d_5_ (RvD2-d_5_) as internal standard (IS) for resolvins, PDx and 7-MaR1. Furthermore, the liquid-liquid extraction (LLE) procedure described proved not to be suitable for skin samples and therefore, SPE was used. The SPE procedure employed was modified from Weiss et al. [31]. Five milling balls, 500 μL of ice cold methanol and 20 μL of a methanolic solution of IS (400 ng/mL) were added to a polypropylene tube containing about 20 mg skin tissue, previously homogenized manually in a mortar with pestle, both chilled in liquid nitrogen. In a Mixer Mill (Mixer Mill MM 400, Retsch, Haan, Germany) the samples were further ground for 4 min at 25 Hz. Then the samples were centrifuged for 5 min at 1680 x g (Biofuge 15 centrifuge, Heraeus Holding GmbH, Hanau, Germany). The supernatant was transferred to an amber glas vial and methanol (LC-MS grade from Roth, Karlsruhe, Germany) was removed under a nitrogen stream at 45°C. Then 1 mL water (LC-MS grade from Roth, Karlsruhe, Germany) was added to the vial. The solution was loaded on a Chromabond^®^ C18/500 mg SPE column (Macherey-Nagel, Düren, Germany), prewashed with 2 mL 90% methanol and 2 mL 5% methanol. After sample loading, the SPE column was washed with 2 mL water, 3 mL 5% methanol and the analytes were eluted with 1.5 mL 90% methanol. The eluate was dried under a nitrogen stream at 45°C and redissolved in 50 μL chromatographic solvent.

### Method Validation

To evaluate the acceptability of the changes in IS and extraction procedure and the appropriateness of the complete assay applied to the determination of SPM in skin as biological matrix, the method was validated in terms of: linearity and calibration range, sensitivity, precision and accuracy, selectivity, recovery, matrix effects and stability, according to the guidelines of the Food and Drug Administration [32] and the International Conference on Harmonisation [33] and as described by Matuszewski et al. [34]. Due to the extensive requirement for skin and consequently mice, the suitability of the assay was assessed at two different concentration levels, corresponding to a low (LLOQ) and medium concentration level (160 pg/mg). Linearity and calibration range, selectivity, as well as precision and accuracy were assessed with spiked skin samples of three different animals, the stability of the analytes in different storage conditions with spiked samples of two different animals and recovery with skin samples of one animal.

#### Validation samples

To 5 mg of skin from the shaved back of untreated mice, 20 μL of the IS (400 ng/mL) and 20 μL of working solutions varying in concentration from 1 ng/mL to 100 ng/mL were added, to give a concentration range of 4.0 pg/mg to 400.0 pg/mg. These samples were extracted according to the SPE procedure outlined in the main manuscript.

#### Linearity and calibration range

Calibration curves (n = 3) covering a range from LLOQ (4 pg/mg for 15-epi-LXA_4_, 17-epi-RvD1, RvD2 and PDx, 8 pg/mg for LXA_4_, 6-epi-LXA_4_, LXA_5_, 7-MaR1, 12 pg/mg for RvD1 and 20 pg/mg for LXB_4_) to 400 pg/mg, of the analytes in the matrix, were prepared and analysed in different days. Calibration curves consisted of a blank sample (matrix sample processed without analytes or IS), a zero sample (matrix sample processed without analytes but with IS) and 7 to 10 non zero samples, depending on the sensitivity of each analyte: 7 non-zero samples for LXB_4_ (20, 30, 40, 100, 200, 300, 400 pg/mg), 8 non-zero samples regarding RvD1 (12, 20, 30, 40, 100, 200, 300, 400 pg/mg), 9 for LXA_4_, 6-epi-LXA_4_, LXA_5_ and 7-MaR1 (8, 12, 20, 30, 40, 100, 200, 300, 400 pg/mg) and 10 for 15-epi-LXA_4_, 17-epi-RvD1, RvD2 and PDx (4, 8, 12, 20, 30, 40, 100, 200, 300, 400 pg/mg). The linearity of each standard line was confirmed by plotting the peak area ratios (analyte/IS) versus analyte concentrations. The analyte concentrations were calculated from the equation y = mx+b, as determined by weighted (1/x) linear regression of the standard line. The absence of the analytes in blank and zero samples was visually evaluated.

#### Lower Limit of Quantitation

The sensitivity of the assay was monitored employing the LLOQ. This was defined as the lowest quantity of analyte which can be quantitated with suitable precision and accuracy and calculated as 10 times the signal to noise ratio of the blank samples in the calibration curves (n = 3).

#### Selectivity

The selectivity of the developed method was tested by comparing the signals obtained for each analyte (peak area) in zero samples at the retention time corresponding to the analyte peak with those obtained from samples spiked with the analyte at a concentration corresponding to the LLOQ.

#### Precision and Accuracy

Intraday precision and accuracy were assessed at two different concentrations, corresponding to a low (4 pg/mg for 15-epi-LXA_4_, 17-epi-RvD1, RvD2 and PDx, 8 pg/mg for LXA_4_, 6-epi-LXA_4_, LXA_5_, 7- MaR1, 12 pg/mg for RvD1 and 20 pg/mg for LXB_4_) and medium (160 pg/mg) concentration level, by extraction and replicate analysis of three spiked samples on the same day. This procedure was repeated on three different days in order to evaluate interday precision and accuracy. As a measure of precision, the percentage relative standard deviation (% RSD) was employed. This is defined as: (SD/mean concentration value) x 100, where SD is the standard deviation of the measurements. The accuracy was evaluated in terms of relative error (RE), the deviation of the calculated concentration from the spiked concentration, expressed in percentage value. This was calculated as: ((calculated concentration-nominal concentration)/nominal concentration) x 100.

#### Stability

Stability of the analytes was tested under different conditions reflecting actual sample treatment and analysis in the laboratory. Three aliquots of each concentration level in spiked samples were extracted after several manipulations and analytes were quantified against freshly prepared calibration curves.

For freeze-thaw stability, the samples were spiked and immediately frozen after preparation at -80°C for at least 24 hours and thawed at room temperature. This procedure was repeated three times, before the samples were extracted and analyzed.

The short-term temperature stability was determined by spiking the samples and maintaining them for 4 hours at room temperature before sample extraction and analysis.

To assess long-term stability, one set of three samples was stored for a period of 14 days and a second set of three samples for a period of 30 days at -80°C before extraction and analysis.

For the determination of the autosampler stability of the analytes, these were kept after extraction and reconstituted in solvent at 7°C for 24 h.

#### Matrix Effects

The matrix effects (ME) were evaluated by comparing the peak area of each analyte at two different concentrations (LLOQ, 160 pg/mg) spiked in skin samples after extraction, to the peak area of an equivalent concentration of the same analyte standard in neat solution. Analyses were repeated three times at each concentration.

Furthermore the influence of matrix effects was studied for all analytes by the comparison of calibration curves (n = 3) in skin to a calibration curve in PBS. For the preparation of the calibration curves in PBS and skin (5 mg/sample), the same working solutions were added in the same amounts to both matrices. To 200 μL of PBS or 5 mg skin, 20 μL of the IS and 20 μL of working solutions varying in concentration from 1ng/mL to 100 ng/mL were added, to give a range of 0.1 ng/mL to 10ng/mL (0.1, 0.2, 0.3, 0.5, 0.75, 1, 2.5, 5, 7.5, 10ng/mL). The slopes of the calibration curves prepared in skin were compared to that in PBS.

#### Recovery

The recovery was determined by the replicate analysis of three sets of three samples each at the low and medium concentration levels of the analytes (LLOQ, 160 pg/mg). Set 1 involved the standards in neat solvent, in set 2 the samples were spiked after extraction and in set 3 the samples were spiked before extraction. In sets 2 and 3, the IS were spiked after the extraction procedure. The relative recovery was calculated as the percentage of analyte which can be extracted from a spiked sample, in comparison to a zero sample, spiked after extraction with the same quantity of analyte. The absolute recovery was calculated as the percentage of analyte, which can be extracted from a spiked sample, in comparison to analyte in neat solvent. For the calculations, the analyte/IS area ratio was employed.

### Statistical analysis

All data are presented as means ± SD, with the exception of the data obtained from the BLI experiment (means ± SEM). To determine statistically significant differences in all experiments, ANOVA for repeated measures was used, followed by Bonferroni’s post hoc correction using GraphPad Prism (GraphPad Software, La Jolla, USA). For BLI measurements comparing only two groups, Student’s t-test was carried out. A confidence interval of 95% and a corresponding p-value of < 0.05 were considered statistically significant.

## Results

### Method Validation for the LC-MS/MS determination of SPM in skin

#### Linearity, calibration range and selectivity

To detect lipoxins and other SPM in skin tissue, we established and validated a method for LC-MS/MS which allows selective and sensitive determination of the SPMs in murine skin.

All calibration curves showed satisfactory linearity for all analytes with correlation coefficient (R^2^) values higher than 0.99. Precision values of the standard line slopes were less than 14%. More detailed information on the validation calibration curves is given in Table 1. The sensitivity of the assay was monitored employing the LLOQ. In all cases, RSD values were lower than 11% and RE values lower than 16%. The selectivity of the developed method was tested comparing the signals obtained for each analyte (peak area) in zero samples at the retention time corresponding to the analyte peak with those obtained from samples spiked with the analyte at a concentration corresponding to the LLOQ. The peak area in the zero samples was always less than 6%, with the exception of 15-epi-LXA_4_ (7.72%) and PDx (8.97%) (Table 1).

**Table 1:** Slope, correlation coefficient, LLOQ and selectivity values obtained for each analyte (n = 3).

Calibration curve parameters					LLOQ			Selectivity
	Average slope	%RSD	Average R^2^	%RSD	pg/mg	%RSD	%RE	%
**LXA5**	0.0100	3.69	0.9973	0.1	8	2.6	15.4	2.0
**15-epi-LXA4**	0.0174	13.0	0.9986	0.1	4	4.3	15.0	7.7
**6-epi-LXA**_**4**_	0.0114	5.26	0.9963	0.2	8	3.8	4.8	1.6
**LXB4**	0.0054	4.95	0.9963	0.1	20	7.5	6.7	3.0
**LXA**_**4**_	0.0260	5.53	0.9984	0.1	8	2.8	15.3	1.4
**17-epi-RvD1**	0.0507	5.12	0.9968	0.2	4	6.2	7.7	0.9
**RvD1**	0.0424	5.40	0.9985	0.1	12	2.4	12.4	0.9
**RvD2**	0.1057	2.01	0.9985	0.1	4	8.8	10.2	5.7
**PDx**	0.0542	5.92	0.9975	0.1	4	4.8	10.5	9.0
**7-MaR1**	0.0134	13.84	0.9986	0.1	8	10.4	9.1	1.8

#### Precision and Accuracy

The suitability of the method has been demonstrated in terms of intra- and interday precision and accuracy, for all analytes. According to the FDA guideline. a value of 20% RSD and RE can be tolerated at the concentration of the LLOQ and 15% RSD and RE for the other concentrations tested. Albeit for inter-individual variations, all values determined were lower. The intraday precision (% RSD) was in all cases lower than 10%. The intraday accuracy (% RE) of the assay ranged from 2.0 to 17.5% for LXA_5_ at the concentration of the LLOQ. For interday precision, the % RSD values obtained were lower than 7% and for interday accuracy, values ranged from 4.4 to 14.2% RE % (Table 2).

**Table 2:** Intraday and interday precision and accuracy values of the analytes at the two studied concentrations, measured on three different days (n = 3).

Analyte	nominal concen-tration (pg/mg)	Intraday precision and accuracy (n = 3)						Interday precision and accuracy (n = 3)	
		%RSD			%RE (mean ± SD)			% RSD	%RE (mean ± SD)
		Animal 1	Animal 2	Animal 3	Animal 1	Animal 2	Animal 3		
	8	0.9	1.0	1.8	17.5 ± 1.0	16.8 ± 1.1	8.3 ± 4.7	1.2	14.2 ± 5.1
**LXA**_**5**_	160	2.9	2.8	2.6	5.4 ± 3.0	8.0 ± 3.0	4.5 ± 2.3	2.7	6.0 ± 1.8
**15-epi-**	4	0.1	4.8	2.5	1.1 ± 0.1	2.3 ± 1.2	10.0 ± 0.1	2.5	5.3 ± 4.0
**LXA**_**4**_	160	2.3	2.3	3.6	3.8 ± 1.0	6.6 ± 2.4	7.8 ± 3.9	2.7	6.0 ± 2.1
**6-epi-**	8	7.2	2.8	3.5	6.7 ± 2.5	6.3 ± 4.8	7.7 ± 0.0	4.5	6.9 ± 0.7
**LXA**_**4**_	160	3.7	1.0	1.7	4 ± 3.3	3.8 ± 1.0	6.8 ± 3.7	2.1	4.9 ± 1.6
	20	3.2	7.3	8.4	11.2 ± 3.5	7.3 ± 3.3	6.8 ± 5.6	6.3	8.4 ± 2.4
**LXB**_**4**_	160	6.5	4.1	1.8	8.3 ± 3.6	7.6 ± 4.4	6.0 ± 3.5	4.1	7.3 ± 1.2
	8	1.0	6.1	1.6	10.0 ± 0.9	5.5 ± 0.9	16.0 ± 1.8	2.9	10.5 ± 5.3
**LXA**_**4**_	160	1.9	3.0	6.7	4.1 ± 2.0	7.3 ± 0.1	4.8 ± 1.0	3.9	5.4 ± 1.7
**17-epi-**	4	3.8	0.5	6.5	3.9 ± 2.2	16.3 ± 0.6	10.3 ± 0.0	3.6	10.2 ± 6.2
**RvD1**	160	3.3	1.5	3.4	5.0 ± 3.9	2.0 ± 1.6	5.6 ± 2.3	2.7	4.2 ± 1.9
	12	2.5	3.0	4.0	8.4 ± 7.0	4.2 ± 3.2	14.8 ± 0.0	3.2	9.2 ± 5.3
**RvD1**	160	4.6	1.7	4.0	5.8 ± 4.3	4.7 ± 0.9	6.0 ± 4.3	3.4	5.5 ± 0.7
	4	2.5	1.4	7.4	7.0 ± 2.7	8.3 ± 3.1	14.0 ± 5.3	3.7	9.8 ± 3.7
**RvD2**	160	0.6	1.8	7.3	10.6 ± 0.6	3.1 ± 0.5	7.2 ± 0.5	3.2	7.0 ± 3.8
	4	2.0	1.0	4.1	6.7 ± 2.1	14.7 ± 1.2	15.7 ± 4.0	2.3	12.3 ± 5.0
**PDx**	160	8.5	0.4	4.0	7.7 ± 0.5	2.4 ± 0.4	3.0 ± 1.5	4.3	4.4 ± 2.9
	8	2.3	4.5	1.5	8.2 ± 7.0	10.0 ± 5.0	18.0 ± 1.8	2.8	12.1 ± 5.3
**7-MaR1**	160	5.8	2.3	9.7	7.8 ± 5.1	9.5 ± 3.2	7.8 ± 5.0	6.0	8.4 ± 1.0

#### Stability

The values obtained after three freeze/thaw cycles ranged from 1.5 to 13.2% RE, while after maintenance of the analytes for 4 h at room temperature all calculated values were lower than 10% RE. All analytes were shown to be stable after storage at -80°C for 30 days, with the exception of 7-MaR1, for which the calculated values were higher than 40% RE. 7-MaR1 proved to be stable after storage for 14 days at -80°C, whereby the calculated values were 9.3 ± 4.5% RE (at 20.0 pg/mg) and 7.8 ± 5.1% RE (at 100 pg/mg). Also after extraction and reconstitution in solvent, the analytes proved to be stable in the autosampler at 7°C for 24 h. All values for each analyte are collected in Tables 3 and 4.

**Table 3:** Stability of samples spiked with different concentrations of the analytes under different storage conditions (n = 2).

Analyte	Concentration (pg/mg)	Short-term stability 4h at room temperature (n = 3)		Long-term stability (30 days) (n = 3)	
		%RE (mean ± SD)		%RE (mean ± SD)	
		Animal 1	Animal 2	Animal 1	Animal 2
	20	0.9 ± 0.5	3.0 ± 0.4	1.7 ± 0.8	3.3 ± 0.7
**LXA5**	100	3.9 ± 1.6	3.6 ± 1.1	1.4 ± 0.5	2.5 ± 0.6
**15-epi-**	12	6.2 ± 4.6	4.8 ± 3.5	7.8 ± 5.8	3.3 ± 2.5
**LXA**_**4**_	40	7.8 ± 2.2	7.4 ± 5.7	4.0 ± 0.9	3.5 ± 2.4
**6-epi-**	20	6.2 ± 1.6	3.6 ± 1.9	6.2 ± 4.3	1.3 ± 0.5
**LXA**_**4**_	100	7.8 ± 3.8	5.7 ± 1.3	8.5 ± 6.1	8.3 ± 4.8
	40	7.9 ± 3.2	9.7 ± 1.0	5.0 ± 2.0	17.8 ± 1.5
**LXB**_**4**_	300	9.3 ± 2.0	7.1 ± 3.4	4.6 ± 3.5	7.4 ± 5.4
	20	2.4 ± 0.6	1.7 ± 0.5	5.8 ± 2.8	6.3 ± 2.4
**LXA**_**4**_	100	3.5 ± 1.9	5.1 ± 1.7	11.9 ± 1.8	9.4 ± 1.1
**17-epi-**	12	4.4 ± 1.8	3.5 ± 1.4	7.2 ± 5.6	7.5 ± 5.2
**RvD1**	40	4.7 ± 3.4	9.6 ± 0.6	8.5 ± 1.0	13.0 ± 1.4
	30	3.8 ± 2.6	4.0 ± 2.4	3.1 ± 1.4	8.2 ± 3.0
**RvD1**	200	6.3 ± 1.0	0.9 ± 0.2	7.2 ± 1.4	6.2 ± 5.0
	12	1.8 ± 0.5	5.6 ± 3.5	4.7 ± 4.5	7.9 ± 6.2
**RvD2**	40	1.4 ± 0.9	4.0 ± 1.0	2.1 ± 1.4	12.5 ± 1.8
	12	4.1 ± 0.7	6.5 ± 2.9	0.7 ± 0.5	6.7 ± 5.4
**PDx**	40	2.4 ± 1.1	2.3 ± 1.7	4.6 ± 3.1	4.9 ± 0.6
	20	0.8 ± 0.4	6.5 ± 3.3	34.7 ± 4.2	41.5 ± 6.4
**7-MaR1**	100	6.1 ± 3.0	6.8 ± 3.6	45.2 ± 7.2	37.9 ± 2.7

**Table 4:** Stability of samples spiked with different concentrations of the analytes after three freeze/thaw cycles and after extraction and reconstitution in solvent in the autosampler (n = 2).

Analyte	Concentration (pg/mg)	Freeze-thaw stability after 3 cycles (n = 3)		Autosampler stability (24h) (n = 3)	
		%RE (mean ± SD)		%RE (mean ± SD)	
		Animal 1	Animal 2	Animal 1	Animal 2
	8	9.0 ± 1.3	5.7 ± 4.8	3.1 ± 2.1	1.0 ± 0.8
**LXA**_**5**_	160	12.0 ± 2.9	2.1 ± 0.7	4.0 ± 2.0	2.1 ± 1.1
**15-epi-**	4	5.7 ± 3.6	10.6 ± 7.4	11.2 ± 2.3	11.6 ± 4.2
**LXA**_**4**_	160	6.8 ± 4.7	4.1 ± 3.0	4.0 ± 3.0	2.1 ± 1.4
**6-epi-**	8	9.1 ± 5.2	7.0 ± 3.2	4.6 ± 1.5	5.2 ± 1.6
**LXA**_**4**_	160	5.0 ± 2.8	1.5 ± 0.5	4.2 ± 2.5	5.1 ± 2.5
	20	11.9 ± 2.1	9.3 ± 4.6	6.3 ± 1.4	13.3 ± 3.0
**LXB**_**4**_	160	7.9 ± 0.9	4.2 ± 2.9	2.7 ± 1.5	4.0 ± 0.4
	8	5.9 ± 2.8	6.1 ± 1.4	5.5 ± 1.6	5.2 ± 1.1
**LXA**_**4**_	160	8.7 ± 6.7	4.4 ± 0.9	6.4 ± 1.5	9.3 ± 1.8
**17-epi-**	4	12.1 ± 7.0	1.6 ± 0.6	7.7 ± 5.6	2.3 ± 1.5
**RvD1**	160	9.9 ± 3.9	6.6 ± 3.8	10.5 ± 2.2	5.5 ± 0.5
	12	4.4 ± 3.0	13.2 ± 2.8	6.2 ± 2.9	7.8 ± 5.8
**RvD1**	160	1.4 ± 0.7	6.2 ± 4.5	6.6 ± 4.6	3.9 ± 0.7
	4	5.8 ± 2.6	10.5 ± 6.3	10.7 ± 5.4	3.1 ± 1.5
**RvD2**	160	9.0 ± 6.0	8.8 ± 1.0	2.9 ± 1.1	3.5 ± 2.6
	4	5.6 ± 2.3	11.7 ± 6.6	1.4 ± 1.0	7.9 ± 1.8
**PDx**	160	7.2 ± 4.1	7.5 ± 3.4	2.8 ± 0.4	3.3 ± 2.0
	8	7.1 ± 1.6	4.6 ± 2.4	6.8 ± 3.3	11.5 ± 0.9
**7-MaR1**	160	3.2 ± 1.0	3.7 ± 1.9	10.4 ± 1.9	3.5 ± 0.7

#### Matrix Effects

The matrix effects (ME) were evaluated by comparing the peak area of each analyte at two different concentrations (LLOQ, 160 pg/mg), spiked in skin samples after extraction, to the peak area of an equivalent concentration of the same analyte standard in neat solution. The calculated values as % ME were about 82 to 109%, with the exceptions of 6-epi-LXA_4_ (68% at 8 pg/mg and 76% at 160 pg/mg) and LXB_4_ (76% at 20 pg/mg) (Table 5).

**Table 5:** Determination of Absolute and Relative Recoveries and Matrix Effects (n = 1).

Analyte	concentration (pg/mg)	Relative Recovery (%)	Absolute Recovery (%)	Matrix Effects (Matuszewski) (% ME)
	8	89.5 ± 3.4	89.2 ± 3.4	83.6 ± 10.2
**LXA**_**5**_	160	86.2 ± 5.0	89.6 ± 5.2	91.5 ± 2.8
**15-epi-**	4	96.6 ± 4.4	100.3 ± 4.5	91.2 ± 9.3
**LXA**_**4**_	160	80.0 ± 1.5	81.8 ± 4.0	87.8 ± 8.6
**6-epi-**	8	88.8 ± 6.6	72.3 ± 5.4	68.1 ± 12.9
**LXA**_**4**_	160	88.0 ± 4.7	73.6 ± 3.9	76.1 ± 9.6
	20	91.7 ± 13.4	97.5 ± 10.6	75.5 ± 13.1
**LXB**_**4**_	160	81.4 ± 2.0	86.4 ± 2.5	90.5 ± 8.5
	8	94.6 ± 2.8	90.6 ± 2.7	99.1 ± 6.7
**LXA**_**4**_	160	71.6 ± 8.6	79.3 ± 9.5	87.6 ± 6.1
**17-epi-**	4	88.8 ± 9.3	94.2 ± 9.8	96.4 ± 13.1
**RvD1**	160	83.9 ± 4.4	89.9 ± 4.7	101.5 ± 10.9
	12	93.3 ± 2.4	94.1 ± 2.4	109.0 ± 9.2
**RvD1**	160	93.1 ± 8.2	89.0 ± 7.8	86.9 ± 13.1
	4	97.1 ± 6.3	98.0 ± 6.4	102.5 ± 8.2
**RvD2**	160	77.3 ± 2.3	82.4 ± 2.5	103.6 ± 10.5
	4	89.6 ± 7.6	98.3 ± 8.4	95.7 ± 8.4
**PDx**	160	82.9 ± 2.0	83.8 ± 2.0	89.0 ± 2.9
	8	92.8 ± 11.5	89.5 ± 11.1	104.3 ± 13.8
**MaR1**	160	72.1 ± 4.3	70.0 ± 4.1	82.3 ± 3.2

**Fig 2:**
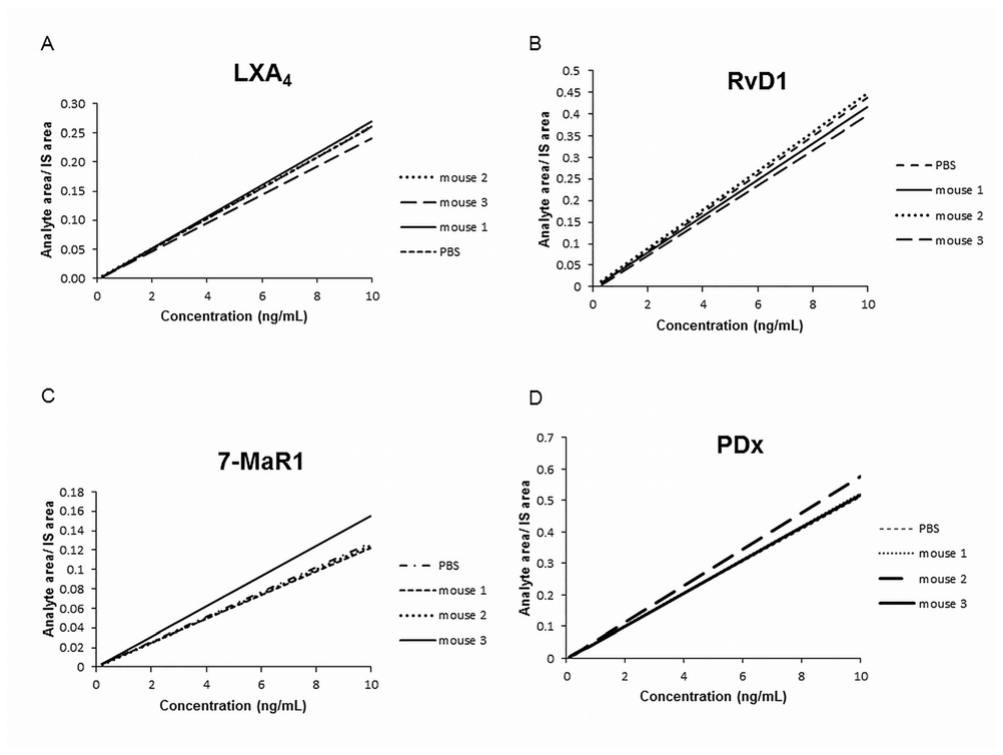
Calibration curves for LXA_4_, RvD1, PDx and 7-MaR1 in skin (n = 3) and PBS. Calibration curves for each analyte were prepared in skin of 3 different mice as well as PBS and compared. Exemplary curves of LXA_4_ (A), RvD1 (B), 7-MaR1 (C) and PDX (D) are shown.

Furthermore, the influence of matrix effects was studied for all analytes by comparison of the slopes of calibration curves (n = 3) in skin to a calibration curve in PBS. For all analytes, the RSD was less than 15%. These results indicate that the quantitation of the analytes in skin samples can be conducted using PBS as an alternative matrix for calibration purposes. All calibration curves for LXA_4_, RvD1, PDx and 7-MaR1 are shown in Fig 2 as examples of each family of SPM.

#### Recovery

The relative recovery was calculated as the percentage of analyte which can be extracted from a spiked sample, in comparison to a zero sample, spiked after extraction with the same quantity of analyte. The values obtained ranged from about 70% (LXA_4_ and 7-MaR1 at 160 pg/mg) to 97% (RvD2 at 160 pg/mg). The absolute recovery was calculated as the percentage of analyte which can be extracted from a spiked sample, in comparison to analyte in neat solvent. For the absolute recovery, the calculated values ranged from about 70% (6-epi-LXA_4_ at 8 and 160 pg/mg) to 100.3% (15-epi-LXA_4_ at 4 pg/mg) (Table 5). For the calculations, the analyte/IS area ratio was employed.

#### Determination of COX-2 expression using qPCR

Number of quantitation cycles was registered for each sample to quantitate COX-2 and the as assay control used proteins (PPIA and RPL 13a). There were no significant differences between the quantitated COX-2 in non-sensitized and sensitized mice, concluding that this enzyme is not affected in the studied model (s. Table 6).

**Table 6:** Quantitation cycles for COX-2, PPIA and RPL 13a measured using qPCR for non-sensitized and sensitized mice (n = 5).

	COX-2		PPIA		RPL13a	
	mean	SEM	mean	SEM	mean	SEM
**non-sens**	32.6	0.2	22.9	0.8	22.5	0.9
**sens**	31.2	0.6	24.5	0.9	25.9	1.1

### Influence of 15-epi-LXA_4_ in skin inflammation

In the OXA-induced dermatitis model, we determined myeloperoxidase (MPO) activities as an indirect measurement of PMN infiltration, using noninvasive and highly sensitive bioluminescence imaging (BLI). The level of the bioluminescence signal slowly increased over 12 h and peaked significantly (P < 0.05) at 24 h and remained constant until 48 h after OXA challenge, after which it rapidly decreased (Fig 1(C)).

To study the influence of the subcutaneous application of 15-epi-LXA_4_ on PMN infiltration in dermal inflammation, we compared the photon flux in the skin area treated with NaCl or 15-epi-LXA_4_ 10 min after the challenge with OXA. The value obtained in the area treated with NaCl was standardized as 100% and the value determined in the area treated with 15-epi-LXA_4_ was related to this value. The photon flux in the area treated with 15-epi-LXA_4_ was significantly lower (P < 0.05) than that in the area treated with vehicle (Fig 1(D)), confirming the known anti-inflammatory effect of 15-epi-LXA_4_.

**Fig 3:**
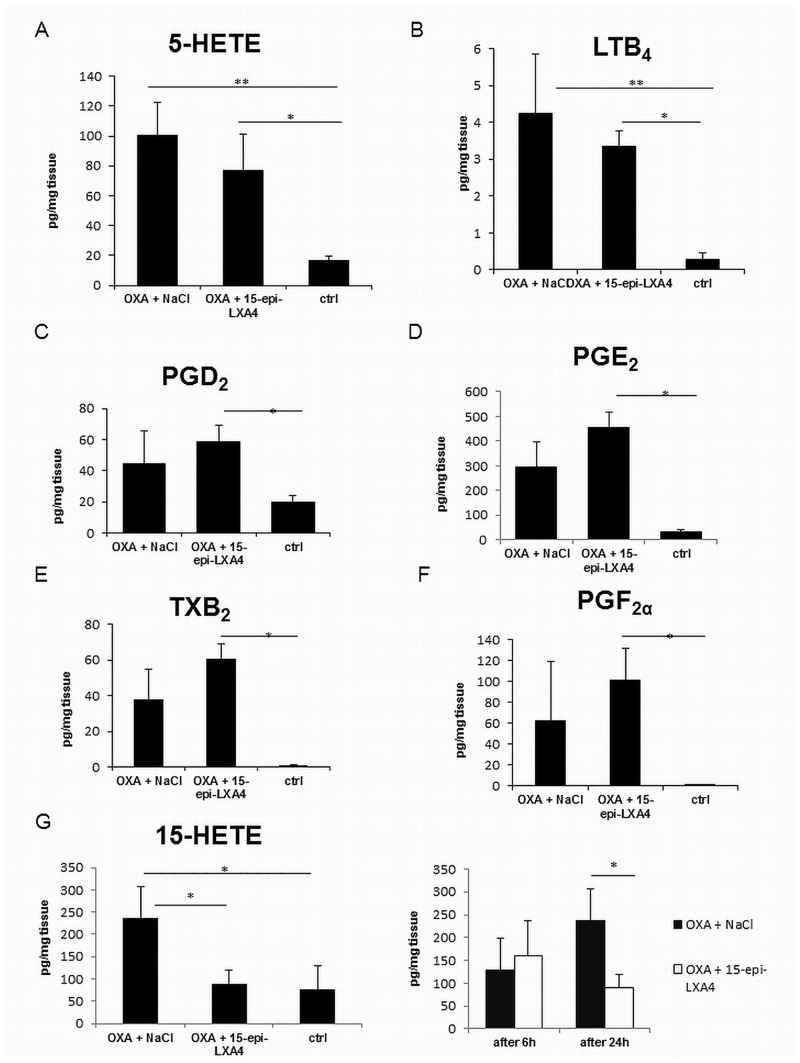
Levels of prostanoids, LTB_4_ and HETE at 24 h after application of 15-epi-LXA_4_ and NaCl. Levels of different prostanoids, products of the COX-pathway, like PGD_2_ (C), PGE_2_ (D), PGF_2α_ (E) and TXB_2_(F), as well as products of the LOX-pathway, such as LTB_4_ (B), its precursor 5-HETE (A) and 15-HETE (G), were determined at 24 h after application of 15-epi-LXA_4_ or NaCl. Concentrations of 15-HETE were also compared 6 and 24 h after application of 15-epi-LXA_4_ or NaCl.

To investigate the biosynthesis of different lipid mediators and their precursors as well as the effects of 15-epi-LXA_4_ on their levels in the OXA-induced dermatitis model, LC-MS/MS analysis was conducted for samples obtained 24h after the challenge with OXA. Since products from the ALOX-pathway (LTB_4_ and its precursor 5-HETE), as well as the prostanoids from the COX-pathway (prostaglandins and thromboxane) are reliable markers for inflammatory responses [35, 36], the levels of 5-HETE, LTB_4_, as well as PGE_2_, PGD_2_, PGF_2α_ and TXB_2_ were quantified in skin treated with OXA and NaCl, OXA and 15-epi-LXA_4_ and untreated control area. 15S-HETE, as a precursor of the lipoxins was also determined. There were no significant differences in the levels of LTB_4_, 5-HETE, PGE_2_, PGD_2_, PGF_2α_ and TXB_2_ when comparing the skin areas treated with OXA and NaCl with those treated with OXA and 15-epi-LXA_4_ (Fig 3). PGE_2_ is reported to be one of the main cutaneous eicosanoids [37]. This was confirmed in the OXA-induced dermatitis model studied here. The calculated concentrations for PGE_2_ were 297.2 ± 176.2 pg/mg skin treated with OXA and NaCl and 454.6 ± 112.8 pg/mg skin treated with OXA and 15-epi-LXA_4_, while PGD_2_ was determined at concentrations of 44.4 ± 20.9 pg/mg skin treated with OXA and NaCl and 58.8 ± 10.43 pg/mg skin treated with OXA and 15-epi-LXA_4_ and the concentrations of PGF_2α_ and TXB_2_ were 63.0 ± 55.9 pg/mg and 38.2 ± 16.8 pg/mg skin treated with OXA and NaCl and 101.2 ± 31.0 pg/mg and 60.9 ± 8.2 pg/mg skin treated with OXA and 15-epi-LXA_4_ respectively. Interestingly, the concentration of 15S-HETE, a precursor of the lipoxins, which are reported to have anti-inflammatory and pro-resolving properties, was significantly (P < 0.05) increased in the skin areas treated with OXA and NaCl (237.4 ± 70.1 pg/mg), compared with the areas treated with OXA and 15-epi-LXA_4_ (89.3 ± 29.8 pg/mg) and the untreated control areas (75.8 ± 29.8 pg/mg) 24 hours after challenge. There was no significant difference between these values and the 15-HETE concentrations measured 6 hours after challenge (129.2 ± 69.9 pg/mg for skin areas treated with OXA and NaCl and 160.8 ± 76.2 pg/mg for skin areas treated with OXA and 15-epi-LXA_4_). However, pro-resolving lipid mediators (lipoxins, resolvins, maresins or protectins) could not be detected in inflamed skin.

### Stability and Distribution of SPM in murine skin after subcutaneous injection

Since the precursor of lipoxins was increased in the skin but lipoxins themselves, and other SPM, were undetectable *in vivo*, we investigated whether the availability or distribution of SPMs was responsible for the lack of analytical signal.

Subcutaneous injection is susceptible to variations in penetration depth, injection speed and pressure, especially in different animals. Therefore, the quantity of each analyte determined by LC-MS/MS in samples gathered directly after injection, was standardized as 100%. For the evaluation of the stability, the amounts detected in samples collected thirty minutes and two hours after injection were related to this value.

**Fig 4:**
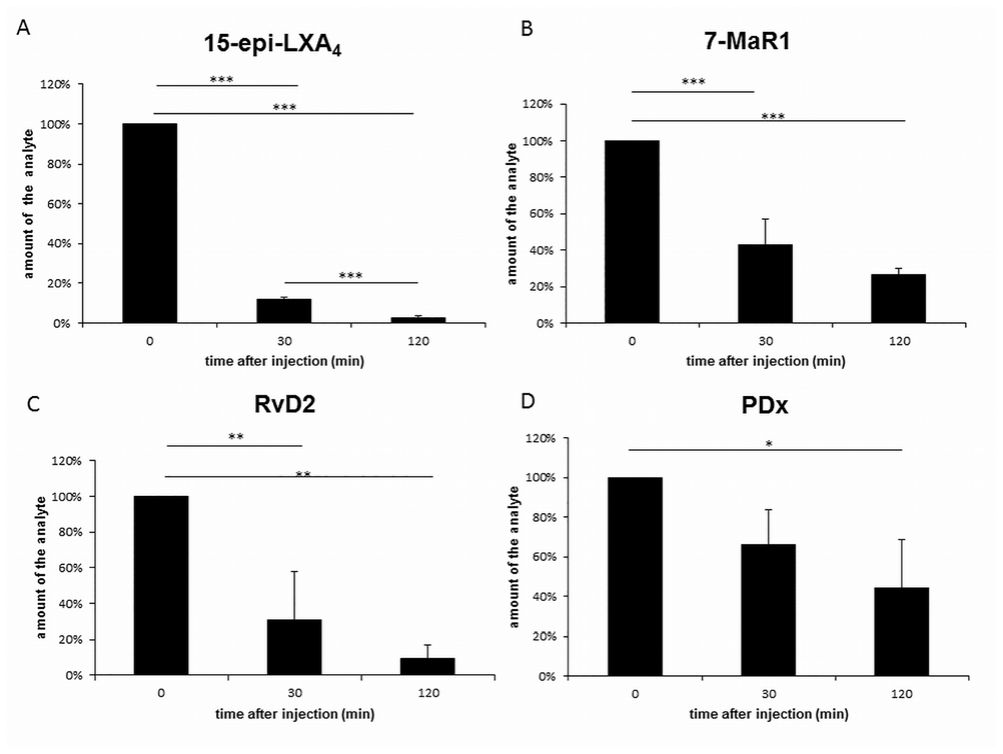
*In vivo* availability of 15-epi-LXA_4_, RvD2, PDX and 7-MaR1. Availability of the SPM was determined by their direct analysis, 30 min and 2 h after their subcutaneous injection into the back skin of the mice. The results for 15-epi-LXA_4_ (A), 7-MaR1 (B), RvD2 (C) and PDX (D) are shown as examples.

Two hours after injection of the SPM, all analytes could still be detected and quantified, although their levels were significantly decreased. The amount of the lipoxins was reduced to 2% (LXA_5_), 3% (15-epi- and 6-epi-LXA_4_) and 4% (LXA_4_ and LXB_4_) (P < 0.001). For RvD1 and its epimer 17-epi-RvD1 values of 38% and 30% were determined (P < 0.001). Only 9% of the injected RvD2 could be recovered. The quantity of 7-MaR1 was reduced to 26% (P < 0.001) and PDx proved to be the most stable of all analytes with a residual amount of 44% (P < 0.05). Already 30 min after injection, the decrease was significant for most of the analytes. The observed reduction in lipoxins was about 88% (15-epi-LXA_4_, 6-epi-LXA_4_, LXA_5_), 81% (LXA_4_) and 71% (LXB_4_) and that in 7-MaR1 about 57% (P < 0.001). The quantity of RvD2 was reduced by 69% (P < 0.01) while for 17-epi-RvD1 and RvD1 a loss of 31% (P < 0.01) and 16% was calculated. In contrary to the rest of the analytes, the decrease in the amount of RvD1 and PDx was not significant (Fig 4).

**Fig 5:**
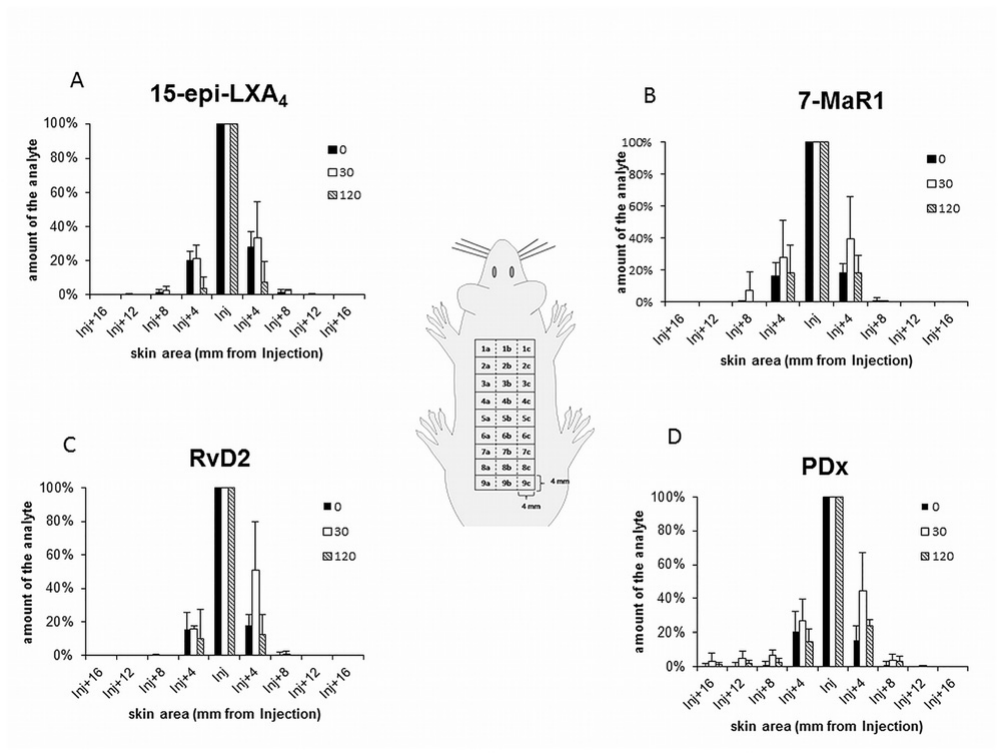
Distribution behavior of 15-epi-LXA_4_, RvD2, PDX and 7-MaR1. The distribution of the SPM was determined by direct analysis of skin areas, 30 min and 2 h after their subcutaneous injection. The results for 15-epi-LXA_4_ (A), 7-MaR1 (B), RvD2 (C) and PDX (D) are shown as examples.

To examine the distribution of the SPMs, the quantities of analytes in each section were compared with the amounts in the corresponding sections directly after injection after 30 and 120 minutes (Fig 5). Sections 4 and 6, vicinal to the section of injection 5, covered the skin area 4 to 8 mm away from the injection point, sections 3 and 7 the skin area 8 to 12 mm away, 2 and 8 the area 12 to 16 mm away and sections 1 and 9 covered the distance from 16 to 20 mm to the location of injection. Directly after injection, lipoxins were detected in sections 3 to 7 (8 to 12 mm away from the injection point), with exception of LXB_4_, which was only detected in sections 4 to 6 (4 to 8 mm away from the injection point). Values ranged from 20 to 35% in the sections 4 and 6, with exception of LXB_4_ for which values of only 10 and 12% were calculated. In the sections 3 and 7 the amounts of the lipoxins ranged from 1 to 4% compared to the amounts in section 5. 15-epi-LXA_4_ was additionally detected in sections 2 and 8 (0.31 and 0.15%). The resolvins were likewise determined in the sections 3 to 7, with exception of RvD2, which was not found in section 3. The values for sections 4 and 6 ranged from 16 to 21% and were 1% in the sections 3 and 7. The values obtained for 7-MaR1 were similar to those of 17-epi-RvD1 (16 and 18% in sections 4 and 6, 0.32% and 1% in sections 3 and 7). The distribution of PDx, directly after injection, differed from that of the other analytes. In addition to the sections 3 to 7 (21 and 16% in sections 4 and 6, 1% in sections 3 and 7), PDx was also present in the sections 1 and 2 (1% in each).

Comparison of the amounts of the SPMs determined in each section after 30 minutes and 2 hours to those obtained directly after injection showed no significant difference in the distribution of the analytes, with the exception of the behavior of 7-MaR1. With regard to section 6, there was a significant difference (P < 0.01) between the amount of 7-MaR1 determined directly after injection (18%) and after 30 minutes (39%). PDx and 17-epi-RvD1 were detected in the same sections as directly after injection (PDx in sections 1–7 and 17-epi-RvD1 in sections 2–7), while LXA_4_, 6-epi-LXA_4_, 15-epi-LXA_4_, RvD1 and RvD2 were not determined in the sections furthest from the injection point already 30 min or 120 min later. LXB_4_ was found directly after injection in sections 4–6, 30 min later additionally in section 7 (5.21%, P > 0.05) and after 120 min only in the section of the injection (section 5) (Fig 5).

## Discussion

The results reported here reveal some new aspects of the field of resolution of dermal inflammation. Despite using a fully validated analytical procedure based on solid phase extraction and LC-MS/MS, we were not able to detect any SPM under physiological conditions in mouse skin. To discern whether the lack of analytical signal was due to the biosynthetic or metabolic/degradation processes, we tried to mimic the production of SPM by injecting them subcutaneously into the skin of the mice. Then we studied the availability of the compounds after injection and their possible distribution in adjacent tissue. Moreover, we studied the role of 15-epi-LXA_4_ in delayed type hypersensitivity and the influence of this compound in the biosynthesis of lipid mediators involved in inflammatory processes.

Several reports on the role of lipoxins in dermal inflammation, suggest that neutrophil-mediated changes in vascular permeability are inhibited by topical application of stable15-epi-LXA_4_ and LXB_4_ analogues, after topical application of acetone and LTB_4_ [22, 23]. In addition, Schottelius et al. examined the role of a 15-epi-LXA_4_-analogue after topical application in various skin inflammation models such as LTB_4_/Iloprost-, croton-oil- and mezerein- induced inflammation [25]. The lipoxin- analogues in most studies were applied topically, with the exception of a publication by Zhang et al., in which native LXA_4_ was repeatedly injectedintraperitoneally, in a PMA-induced acute skin inflammation model [24]. To understand the physiological processes in the resolution of OXA-induced skin inflammation, we examined the influence of 15-epi-LXA_4_, which is reported to be biosynthesized under the influence of acetylated or nitrosylated COX-2 or CYP450-enzymes. 15-epi-LXA_4_ was administered subcutaneously to imitate its local biosynthesis. We observed that MPO activity (used as an indirect measure of PMN infiltration) was significantly increased at 24 h after the challenge with OXA. This finding confirmed those made in various other models of dermal inflammation, in which the maximal neutrophilic infiltrate occurs at the same time point [38–40]. Although, in our model, the MPO activity at 48 h remained comparably high and did not decrease again until 72 h after the induction of the inflammation, the influence of 15-epi-LXA_4_ on the PMN infiltration and likewise on the biosynthesis of pro- as well as anti-inflammatory and pro-resolving lipid mediators was examined after 24 h. We cannot compare the concentrations of prostaglandins, LTB_4_ or various HETE with the inflammatory infiltrate, since these mediators were not analysed in previous publications dealing with dermal inflammation. While 15-epi-LXA_4_ significantly reduced PMN infiltration at 24 h after the OXA-challenge, it did not significantly change the levels of the eicosanoids PGE_2_, PGD_2_, PGF_2α_ and TXB_2_, products of the COX-2 pathway, nor LTB_4_ or as its precursor 5-HETE, biosynthesized under the influence of ALOX. These results suggest that there is no direct relationship between the influence of 15-epi-LXA_4_ and the activity of COX-2 (which expression is not affected in the used animal model, as supported by the qPCR data) or 5-ALOX. These findings confirm the definition of SPM, which are regarded as pro-resolution molecules, and are clearly distinguishable from anti-inflammatory mediators. In sharp contrast to anti-inflammatory processes, resolution processes do not completely inhibit the infiltration of PMN and their activation, nor the interactions between PMN and endothelial cells or the aggregation of platelets. SPMs have no immunosuppressive properties which could lead to infections, rather they promote the return to tissue homeostasis by enhancing the clearance of apoptotic PMN, microbes and cell debris through macrophage phagocytosis [1, 41–43]. In addition, COX-2 and 5-ALOX, in addition to 15-ALOX, are key enzymes in the biosynthesis of the SPM. Additionally, PGE_2_ and PGD_2_, which are mainly regarded as pro-inflammatory lipid-mediators, were reported to promote resolution of inflammation by up-regulating the expression of 15-ALOX [44]. The observations made here reveal that the administration of 15-epi-LXA_4_ reduces PMN infiltration, without negatively influencing other beneficial components of the resolution of inflammation.

LTB_4_ is one of the most potent endogenous stimuli of PMN and participates in their recruitment in pathophysiologic scenarios. The assumption that the influence of 15-epi-LXA_4_ on LTB_4_-synthesis explain the reduced PMN infiltration, could not be confirmed by our data. There was no significant difference between the levels of dermal LTB_4_ after treatment with 15-epi-LXA_4_ or vehicle. Other mechanisms must be involved in the reduction of PMN-infiltration and more studies are necessary to explore the context. Several mechanisms have been proposed to explain the bioactions of lipoxins. These include activation of a G-Protein coupled receptor, later designated as ALX/FPR2 receptor, activation of cysteinyl peptide receptors or cellular uptake of LXA_4_ [45–49]. The human formyl peptide receptor (FPR) family consists of three members, FPR, FPR2/ALX and FPR3 [50]. FPR2/ALX was reported to be expressed in several types of leukocytes such as neutrophils, monocytes, macrophages and activated T-cells among others [51]. It has been described that the interaction of LXA_4_ with this receptor expressed on neutrophils stop their migration [52]. However, these receptors can also be activated by other molecules and some publications question their activation via LXA_4_ [53–56]. In mice, 8 members of the formyl peptide receptor have been described: Fpr1, Fpr2, and Fpr-rs1 (the two lasts have been proposed to bind LXA_4_ [23, 57]) are regarded as orthologues of the human FPRs, while Fpr-rs3, Fpr-rs4, Fpr-rs6, Fpr-rs7 and Fpr-rs8 are exclusive of mice.

Although SPM could not be detected by LC-MS/MS analysis of the skin, either in tissue treated with 15-epi-LXA_4_ or in tissue treated with vehicle, the detection of 15-HETE, a pathway marker for the biosynthesis of lipoxins, provides further insight into inflammation and resolution processes and the effects of 15-epi-LXA_4_ in this model. The levels of 15-HETE were significantly reduced in skin after application of 15-epi-LXA_4_, suggesting that the onset of resolution of inflammation at 24 h after OXA is more advanced after application of 15-epi-LXA_4_. Taken together with the significantly reduced levels of MPO activity at the site of inflammation, the data confirm the role of 15-epi-LXA_4_ as a promoter of the resolution of inflammation in the OXA-induced dermatitis model.

The question remains why SPMs could not be detected. In addition to a functional LC-MS/MS method, the location and the time point of the analysis were identified as determining factors for successful analysis of the SPMs. The suitability of the method was tested during a complete method validation and could be confirmed also for low concentrations of SPM. The LLOQ ranged from 4.0 to 20 pg/mg tissue. SPMs have been reported to be locally biosynthesized at the site of inflammation, but to our knowledge, the distribution potential of these analytes has never been examined. For this purpose, the shaved backs of mice were divided into measured sections and the amounts of LXA_4_, 15-epi-LXA_4_, 6-epi-LXA_4_, LXB_4_, LXA_5_, RvD1, 17-epi-RvD1, RvD2, PDx and 7-MaR1 in these sections were determined directly, 30 min and 2 h after their subcutaneous injection. After subcutaneous injection, intended to imitate local biosynthesis in dermal inflammation, the SPMs did not diffuse away from the point of injection. Thus, on one hand, distribution of the SPMs into adjacent tissues cannot be expected but on the other hand our findings underline the importance of the exact location and the time point of SPM synthesis for successful detection.

The study of the *in vivo* availability of LXA_4_, 15-epi-LXA_4_, 6-epi-LXA_4_, LXB_4_, LXA_5_, RvD1, 17-epi-RvD1, RvD2, PDx and 7-MaR1, after subcutaneous injection revealed some as yet undescribed aspects. Although all SPM decreased significantly at 2 hours after the injection, the DHA-derivatives were more stable than the AA- and EPA-derivatives. Thus might be because the enzymes participating in the metabolism of these compounds have higher affinity to AA-derivatives than to DHA-derivatives. 15-prostaglandin dehydrogenase (15-PGDH), as well as CYP450 enzymes, are known to participate in the metabolism of SPMs [12, 46, 58, 59]. The first step in the metabolism of lipoxins and resolvins, under participation of 15 PGDH, is dehydrogenation hydroxygroups leading to oxo-derivatives. Compared with native lipoxins and resolvins, which are products of the ALOX-pathway, their Aspirin^®^-triggered isomers are metabolically converted at a slower rate, indicating stereospecifity of 15-PGDH [46]. In our stability study in mice, a difference between native and Aspirin^®^-triggered isomers in the rate of metabolic conversion was not observed. Two hours after injection, the quantities of LXA_4_ and 15-epi-LXA_4_, as well as RvD1 and 17-epi-RvD1, were nearly the same. Thus, 15-PGDH might not be the main enzyme involved in SPM metabolic inactivation in this special context. The covalent binding of cell membrane phospholipids to proteins [60] or drugs and drug metabolites to tissue [61] have already been described. It is also possible, that covalent binding could have played a role in the lack of free SPMs we observed *in vivo*.

The period during which analysis of SPMs by LC-MS/MS is possible in our dermatitis model is very short compared with the time course of the inflammation and its resolution. In further studies, the analysis will be conducted at additional time points, including those later than 24 h, since the level of 15-HETE, an intermediate in the biosynthesis of the lipoxins was high at 24 h.

In the context of the OXA-induced dermatitis model, 15-epi-LXA_4_ itself proved to be stable for about 2 h, and exerted positive effects in reducing PMN infiltration even at 24 h after application, meaning, that the inhibitory action of 15-epi-LXA_4_ lasts longer than it remains present in the skin. In future studies, this aspect will be considered further.

## Supporting Information

S1 ARRIVE Guidelines Checklist NC3Rs ARRIVE Guidelines Checklist. (PDF)
